# Targeted next-generation sequencing of TP53 in oral tongue carcinoma from non-smokers

**DOI:** 10.1186/s40463-016-0160-4

**Published:** 2016-09-17

**Authors:** Daniel L. Faden, Sarah T. Arron, Chase M. Heaton, Joseph DeRisi, Andrew P. South, Steven J. Wang

**Affiliations:** 1 Department of Otolaryngology-Head and Neck Surgery, University of California, 2380 Sutter St First Floor, San Francisco, CA 94115 USA; 2 Department of Dermatology, University of California, San Francisco, CA USA; 3 Department of Biochemistry and Biophysics, University of California, San Francisco, CA USA; 4 Department of Dermatology and Cutaneous Biology, Thomas Jefferson University, Philadelphia, PA USA

**Keywords:** Oral tongue carcinoma, TP53

## Abstract

**Background:**

Little is known regarding the etiology and genomic underpinnings of Oral Tongue Squamous Cell Carcinoma (OTSCC) in patients who lack traditional risk factors, yet the incidence is increasing. In particular, the rate, and role, of TP53 mutations in this cohort has been heavily debated in the literature.

**Methods:**

Tumor DNA from forty-three non-smokers with OTSCC underwent next generation sequencing of TP53.

**Results:**

Sixty percent of samples contained a TP53 mutation. The G > T transversion rate was 5.7 %. TP53 status did not differ by age.

**Conclusions:**

OTSCC in non-smokers have TP53 mutation rates similar to other Head and Neck cancers yet these mutations do not appear related to carcinogen exposure based on the mutational spectrum and clinical history. The mechanisms driving tumorigenesis in this cohort, including mutations in TP53, remain elusive and further studies are needed.

**Electronic supplementary material:**

The online version of this article (doi:10.1186/s40463-016-0160-4) contains supplementary material, which is available to authorized users.

## Introduction

Oral Tongue Squamous Cell Carcinoma (OTSCC) most commonly occurs in older patients who smoke and drink. The incidence of OTSCC is decreasing in the United States as the rates of tobacco use in the population decrease. However, OTSCC in young patients (<45) is increasing in incidence in the United States and does not appear to be associated with the known risk factors for head and neck squamous cell carcinoma (HNSCC) including tobacco/alcohol abuse, infection with human papilloma virus (HPV), or any other virus [1, 2]. Little is known regarding the genomic underpinnings of OTSCC in patients who lack traditional risk factors. It remains unclear if this cohort is genomically and etiologically distinct from traditional OTSCC.

TP53 (p53) mutations are the most common mutations in HNSCC and appear to play a pivotal role in tumor initiation. The rate of p53 mutations in non-smokers, particularly OTSCC in young patients, is highly variable in the literature [1, 3, 4]. In order to help shed light on this important question, we performed targeted Next Generation Sequencing (NGS) on OTSCC samples from forty-three non-smokers.

## Methods

After histologic assessment, tumor DNA was extracted from FFPE tissue sections (QIAamp DNA FFPE Tissue Kit, Qiagen, Hilden, Germany) from forty-three patients with surgically treated OTSCC. All patients were never smokers and had no history of radiation exposure or chemotherapy. 454 Pyrosequencing of p53 was performed using the GS Junior system (Roche/454 Life Sciences, Branford, CT) and Fluidigm (Fluidigm Corporation, San Francisco, CA) PCR amplicon libraries as template according to recommended guidelines (Roche, Mannheim, Germany). 454 variant detection required a minimum of four supporting reads and a minimum variant allele frequency threshold of 0.1. Samples with coverage less than 20 % at 10x were filtered out to ensure accurate mutation frequency calculations.

## Results

Thirty-six samples had adequate sequencing coverage for inclusion (Additional file 1: Table S1). The mean age was 58 years old. Eight patients were under 45. Forty-five variants in p53 were identified in 22 different samples (60 % of samples) (Additional file 1: Table S2), including the common polymorphism Pro72Arg. Thirty-four of these variants (53 % of samples) were known or predicted to be deleterious. The rate of deleterious mutations in patients <45 and >45 was 50 % and 58 %, respectively. The mutational spectrum in order of frequency was the following: G > A 45.7 %, T > G 20.0 %, T > C14.2 %, G > C 11.4 %, G > T 5.7 %, T > A 2.8 %. Six samples contained a missense mutation at codon 382 (Lys382Asn).

## Discussion

OTSCC in non-smokers, particularly young patients, is an intriguing cohort as they do not have the traditional HNSCC risk factors, and are increasing in incidence in the United States [1, 2]. Many of these tumors also appear to lack key driver mutations present in other HNSCC, suggesting they are more than just epidemiologically distinct. Little is known regarding the genomic and etiologic underpinnings of these tumors. Highlighting this paucity of knowledge is the considerable variability in reported mutation rates of known cancer causing genes, particularly p53.

p53 functions as a powerful regulator of the cell cycle and apoptosis and is the most commonly mutated gene in HNSCC (47–62 %) [5, 6]. In OTSCC, reported p53 mutation rates are highly variable ranging from 20–94 % [1, 3, 4]. Some of this variability may be related to mutation identification techniques that either under call (Sanger Sequencing of limited high yield exons) or over call (WES without mutation validation) mutations. Other reasons include small cohort sizes, imprecise patient stratification and heterogeneity within the cohorts. Our results suggest that the rate of p53 mutations in OTSCC from non-smokers is similar to other HNSCC, including OTSCC from smokers, with 53 % of samples having a deleterious mutation. If anything, our data may under call the true mutation rate as some select exons had sub-optimal sequencing coverage due to poor DNA quality. Interestingly, six samples contained a common mutation (Lys382Asn). Evidence suggests that acetylation of Lys 382 is required for P53 activation and transcriptional activity [7].

p53 mutations in most tobacco/alcohol associated HNSCC are assumed to be directly related to DNA damage from carcinogen exposure. Examination of the mutational spectrum of a tumor can help elucidate the etiology of mutations. Smoking associated mutations tend to have a preference for G > T (C > A) transversions [8]. Our data show a low (6 %) G > T transversion rate, consistent with other non-smoking related cancers outside the head and neck, and with findings in other oral tongue cohorts [4, 8]. One caveat to consider when assessing the mutational spectrum is that DNA extracted from FFPE can be susceptible to cytosine deamination to uracil as well as 5 methyl cytosine deamination to thymine, which can lead to artefactual C:G > T:A mutations during PCR amplification. Therefore, the percentage of C > T changes could reflect some degree of artifact and should be interpreted knowing this. None-the-less, our data suggests that while p53 mutations in non-smokers with OTSCC are as prevalent as other HNSCC, and likely vital to tumorigenesis, the etiology of these mutations remains unclear.

## Conclusion

While the overall rate of HNSCC and OTSCC is decreasing in the United States, the rate of OTSCC in young patients who do not smoke is increasing. Whether these cancers represent simply an epidemiologically distinct cohort or if they represent a genomically and etiologically distinct cohort remains to be determined. Our data suggests OTSCC in non-smokers have rates of p53 mutations similar to other HNSCC (~55 %) yet these mutations do not appear related to carcinogen exposure based on the mutational spectrum and clinical history. The mechanisms driving tumorigenesis in this cohort, including mutations in p53, remain elusive. Further studies are needed to elucidate the processes driving tumor development.

## Additional file

Additional file 1: Table S1. Demographic data for patient samples included in analysis (coverage >20 % at 10x). **Table S2.** TP53 Mutations. (DOCX 293 kb)

## Abbreviations

OTSCC: Oral tongue squamous cell carcinoma
HNSCC: Head and neck squamous cell carcinoma
HPV: Human papilloma virus
NGS: Next generation sequencing
