# Supplementary material for: Targeted next-generation sequencing of TP53 in oral tongue carcinoma from non-smokers
Source: J Otolaryngol Head Neck Surg. 2016 Sep 17;45:47. doi: 10.1186/s40463-016-0160-4 (PMC5027093; doi:10.1186/s40463-016-0160-4)
Supplement: Additional file 1: Table S1. — Demographic data for patient samples included in analysis (coverage >20 % at 10x). Table S2. TP53 Mutations. (DOCX 293 kb) [file 40463_2016_160_MOESM1_ESM.docx]

**Targeted Next-Generation Sequencing of TP53 in Oral Tongue Carcinoma From Non-smokers**

Daniel L. Faden, MD^1^, Sarah T. Arron MD, PhD^2^, Chase M. Heaton, MD^1^, Joseph DeRisi, PhD^3^, Andrew South, PhD^4^, Steven J. Wang, MD^1^

Table S1. Demographic data for patient samples included in analysis (coverage >20% at 10x)

Table S2. TP53 Mutations
